# Doctors' Wills

**Published:** 1914-02-14

**Authors:** 


### Doctors' Wills.

Mr. John Thomas Hartill, M.R.C.S., L.R.C.P., of Lillenhall, for many years medical officer of health for the town, has left estate of the gross value of £14,052, of which £6,651 is net personalty.

Dr. Josiah William Walker, M.D., L.R.C.S., late resident surgeon at the Edinburgh Maternity Hospital and resident medical officer at the Greenwich Union, left estate of which £18,217 is net personalty.

Mr. Horace Dimock, M.A., M.B., B.C., of Wisbech, surgeon, who died on October 27 while under remand on a charge of libel arising out of differences with local practitioners on the Insurance Act, has left estate of the gross value of £3,519, of which the net personalty has been sworn at £1,831.

Colonel Clement Godson, M.D., V.D., formerly president of the British Gynæcological Society, consulting physician to the City of London Lying-in Hospital and St. Peter's Home, Kilburn, a former Master of the Shipwrights' Company, left estate of the gross value of £48,492, of which £44,633 is net personalty.

